# Burden of Communicable Disease among the Native and Repatriating Afghans

**DOI:** 10.1371/journal.ppat.1002926

**Published:** 2012-10-25

**Authors:** Syed H. Abidi, Farwa Ali, Farida Shah, Farhat Abbas, Syed Ali

**Affiliations:** 1 Department of Biological and Biomedical Sciences, Aga Khan University, Karachi, Pakistan; 2 Nursing & Midwifery, Aga Khan University Program, Kabul, Afghanistan; 3 Department of Surgery, Aga Khan University, Karachi, Pakistan; The Fox Chase Cancer Center, United States of America

Owing to the capricious political and economic conditions of Afghanistan during the past few decades, the burden of communicable disease, especially blood-borne and sexually transmitted infections, has been steadily mounting in this population. Afghan refugees who sought asylum and settled in foreign countries encountered many a barrier to standard health care, which led to the establishment of several epidemics in the refugee population as well. In recent years, repatriation of these refugees back to their homeland has raised new concerns about control and prevention of potential epidemics in the returning and indigenous Afghan populations. Mixing of existing infections, as well as of risk behaviors and practices, in the two populations, if left unattended, can lead to unforeseen, uncontrollable outcomes.

In 2002, the United Nations High Commissioner for Refugees (UNHCR) initiated efforts for repatriation of Afghan refugees to their native country. Since early 2002, more than 5 million Afghan refugees have been repatriated from both Pakistan and Iran back to their native country. In 2007, more than 350,000 refugees were repatriated from Pakistan. Afghanistan's economic sustenance has been dependent on aid from the United States, the International Committee of the Red Cross (ICRC), the World Food Program, and numerous other non-governmental organizations [1]. While Afghanistan struggles to cover all its deficits, devising settlement and support plans for internally displaced peoples (which, according to UNHCR, were 235,833 in 2009) and returning refugees would be too much to ask [2]. At this point, managing the massive influx of repatriates and arranging for their accommodation in urban and rural settings would be an insurmountable challenge for Afghanistan. Although these repatriation efforts are well-intentioned, they appear to be negligent of health care concerns for the returning Afghans. It appears that the burden and geography of existing communicable disease among the refugees are overlooked. Pressures of re-settlement, terrorist and military activities, and political and economic instability are likely to facilitate the proliferation of existing epidemics in the repatriated and indigenous Afghan populations.

In the past few decades, persistent military intervention, the Soviet invasion, terrorism, and Talibanization have forced Afghan refugees to migrate from Afghanistan (Figure 1), with the majority of them settling in Pakistan and Iran [3]. These refugees, some now in their third generation [4], [5], became an active component of the socioeconomic structure, engaging in various jobs, and sharing economic and social benefits with the natives of the host countries. Seeded by the experience of war, insecurity, and terror, refugees suffer from post-traumatic stress disorder, depression, and other mental morbidities [6], [7]. In the developing countries of Iran and Pakistan, where the host population have to struggle for basic necessities of life, issue of mental morbidity is rarely addressed. Although the Afghan government has identified mental health as one of the five major health problems among the Afghans, the country is not yet ready to deal with the issue on a national level [6]. A large number of mentally morbid repatriates, unfit for employment, self-sustenance, or for independently supporting a functional family unit, is likely to aggravate the existing health challenge in Afghanistan.

**Figure 1:**
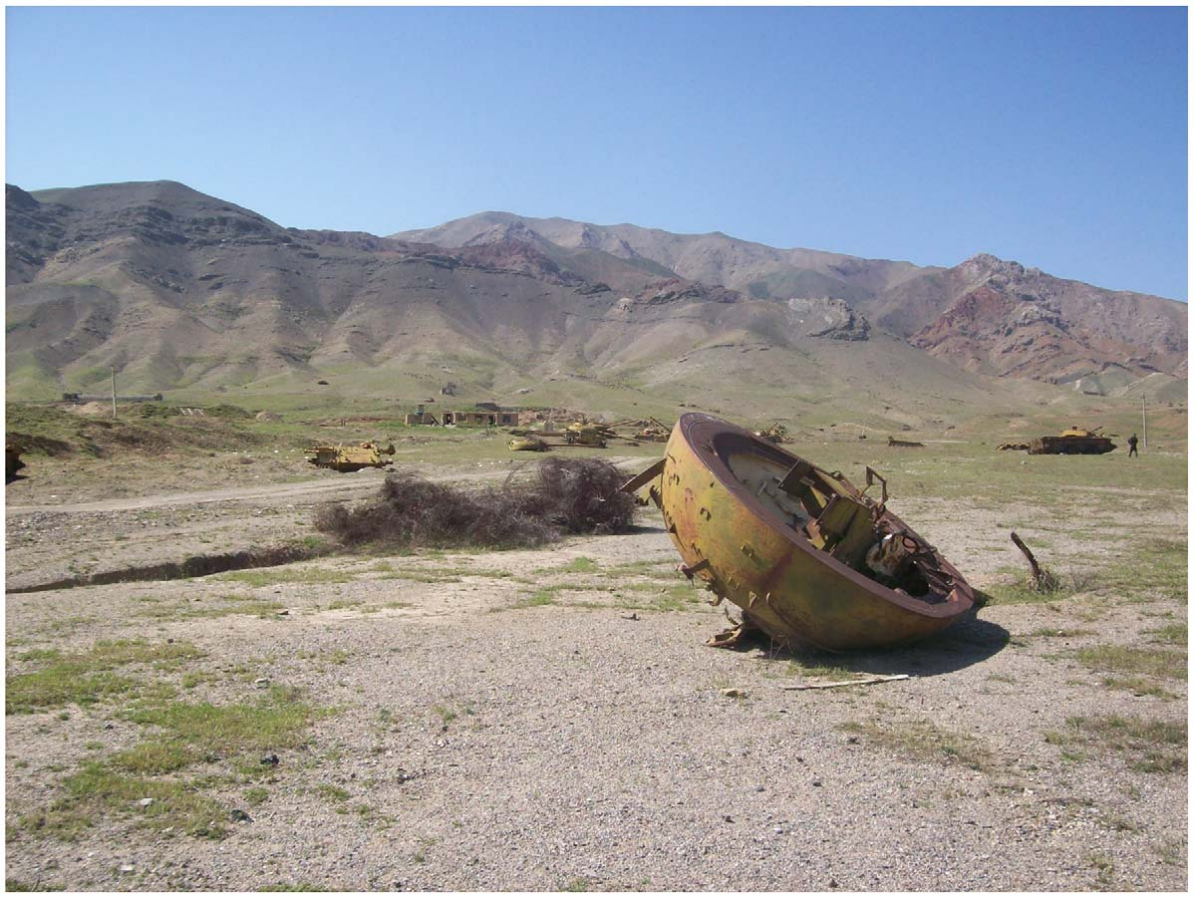
Post-war Afghanistan awaits returning Afghans. The picture shows artillery used during the war in Afghanistan, now rusted and fallen apart in the outskirts of Kabul.

Refugee populations from war-afflicted countries get exposed to certain risk factors that predispose them to sexually transmitted, blood-borne, and other types of infections [10]. During and following the decades of warfare, native and refugee Afghans have constantly struggled with innumerable disadvantages, including illiteracy, low socioeconomic conditions, rampant unemployment, and an overall limitation of life-sustaining resources. Those limitations, combined with inadequate health care, gave rise to an exponential proliferation of infectious diseases, which was aggravated further in refugee colonies, since the preexisting burden of infectious diseases was already heavy in the developing host nations of Pakistan and Iran. The burden of such infections has reached alarming levels in Afghan refugees residing in Pakistan; the reported prevalence being 6%, 9%, and 37% for, respectively, human immunodeficiency, hepatitis B, and hepatitis C viruses (HIV, HBV, and HCV, respectively) [8]. In one study, use of intravenous drugs was reported to be a common risk factor among the Afghans for acquiring HIV. Frequent travel and transmigration, on the other hand, were the main factors that fueled transmission and evolution of novel HIV variants [9].

To date, 556 HIV cases have been reported in Afghanistan; however, the Joint United Nations Programme on HIV/AIDS (UNAIDS) and the World Health Organization (WHO) estimate that this number may be as high as 1,000 to 2,000 [10]. Through sexual as well as vertical transmission, HIV transmission among Afghan children has also been a growing concern [11]. Afghanistan is the world's largest producer of opium, and a 2005 survey estimated that Afghanistan had almost 1 million drug users, including 200,000 opium users and 19,000 drug injectors (of whom 12,000 inject prescription drugs and 7,000 inject heroin). In view of the risk factors, including intravenous drug abuse, usage of contaminated syringes, contact with commercial sex workers, men having sex with men, and a low sexual awareness among Afghan refugee population [8], the high prevalence of sexually transmitted diseases is hardly surprising. Intravenous drug use, transfusion of contaminated blood, and sexually promiscuous behavior have reportedly led to a high prevalence of hepatitis B in Afghanistan as well [12]. The prevalence rate of hepatitis B among Afghan refugees residing in Pakistan and the United States is also considerably high [3], [8], [13]. Despite efforts from government and non-governmental organizations, immunization programs have not been rigorously administered among the refugee communities. As a result, the prevalence of preventable infections, such as HBV, measles, and polio, has inevitably risen in this population [3], [13], [14]. Considering that in Afghanistan, door-to-door vaccination programs are seriously lacking [15], the burden carried by the returning refugees will only amplify in their native land.

## Recommendations

In Pakistan, before repatriation, a complete and through registration of all refugees in all rural and urban settings should be completed so that the refugees are not only easily accessible, but are also grouped into various categories based on their health care needs. This would help in addressing issues faced by each group separately. Families financially well-off or/and with preexisting family links in Afghanistan should be repatriated first so that the government could buy time to devise a support plan for those repatriating without any support. Additionally, in collaboration with international agencies, an emergency assistance plan should be devised to initiate mass screening of diseases and infection in all refugee camps so that the budding epidemics may be caught and controlled in time. The refugees should also be provided psychological counseling to help them deal with mental morbidities. Immunization should be incorporated into the repatriation plan so that the refugees, prior to their return, can be immunized against common infections. To increase their chances of success, the immunization programs should be followed up and strictly adhered to in Afghanistan. Major high risk groups must be targeted for tailored prevention and control programs; needle-exchange and sex education programs should be implemented for injection drug users and for those involved in unsafe sex. Native Afghan men and women should be trained as health workers and educators to administer mass awareness, immunizations, mental counseling, and health screening campaigns. The element of familiarity and improved communication introduced this way will ensure the success of the various health initiatives (Table 1).

**Table 1:** Risks associated with native and refugee Afghans and possible interventions.

Risk	Intervention Recommended
Massive influx of repatriates and IDPs	- Registration of repatriates- Structured repatriation, starting with those having preexisting ties in Afghanistan
Mental morbidity	- Psychological counseling
Communicable disease	- Regularized screening- Immunization
Sexually transmitted disease	- Sex education, awareness
Injection-drug use	- Needle exchange programs
Low awareness	- Health campaigns and counseling by native Afghans

IDPs, internally displaced peoples.
